# Infection prevention and control of *Candida auris* in pediatric settings

**DOI:** 10.1017/ash.2026.10419

**Published:** 2026-06-23

**Authors:** Thomas S. Murray, Hana Hakim, Christelle Ilboudo, Lynn Ramírez-Ávila, Jana Shaw, Terri Stillwell, Patricia Budo, Bernard Ebruke, Noa Fleiss, Amanda Green, Matthew Linam, Roshni Mathew, April McDougal, Natalie Neu, Zoi Dorothea Pana, Karen Ravin, Ayelet Rosenthal, Jane D. Siegel, Katlyn Steimel, Amy Valencia, Carol Vance, Lars F. Westblade, Lisa Saiman

**Affiliations:** 1 Yale New Haven Children’s Hospital, New Haven, CT, USA; 2 Yale School of Medicine, New Haven, CT, USA; 3 St. Jude Children’s Research Hospital, Memphis, TN, USA; 4 Children’s Mercy Kansas City, Kansas City, MO, USA; 5 University of Missouri-Kansas City, Kansas City, MO, USA; 6 University of California-San Francisco, San Francisco, CA, USA; 7 Upstate Medical University of The State University of New York, Syracuse, NY, USA; 8 UPMC Children’s Hospital of Pittsburgh, Pittsburgh, PA, USA; 9 University of Pittsburgh School of Medicine, Pittsburgh, PA, USA; 10 Pediatric Complex Care Association (PCCA), Vancouver, WA, USA; 11 International Foundation Against Infectious Disease in Nigeria (IFAIN), Nigeria; 12 Healthy Child Care Initiatives for Child Care Aware of America, St. Petersburg, FL, USA; 13 Children’s Healthcare of Atlanta, Inc., Atlanta, GA, USA; 14 Emory University School of Medicine, Atlanta, GA, USA; 15 Lucile Packard Children’s Hospital Stanford, Palo Alto, CA, USA; 16 Stanford Medicine Children’s Health, Palo Alto, CA, USA; 17 The University of Texas Medical Branch, Galveston, TX, USA; 18 University of Nicosia Medical School, Cyprus; 19 Nemours Children’s Hospital Delaware, Wilmington, DE, USA; 20 Ann & Robert H. Lurie Children’s Hospital of Chicago, Chicago, IL, USA; 21 Northwestern University Feinberg School of Medicine, Chicago, IL, USA; 22 Retired, San Diego, CA, USA; 23 Advocate Children’s Hospital of Advocate Health, Chicago, IL, USA; 24 Weill Cornell Medicine, New York, NY, USA; 25 Columbia University Irving Medical Center, New York, NY, USA

## Abstract

**Background:**

*Candida auris* (also referred to as *Candidozyma auris*) is an emerging multidrug-resistant fungal pathogen associated with high morbidity and mortality. Existing infection prevention and control (IPC) guidance has largely focused on adult populations, with limited recommendations for pediatric healthcare and non-healthcare settings.

**Methods:**

The Society for Healthcare Epidemiology of America (SHEA) convened a multidisciplinary expert panel to develop IPC recommendations for *C. auris*. The panel developed recommendations using a structured, iterative Delphi consensus process with rounds of discussion, refinement, and anonymous electronic voting with predefined consensus thresholds. Panelists reviewed relevant peer-reviewed and gray literature integrated with expert judgment and practical considerations. Preambles and remarks provide additional context and guidance.

**Results:**

This consensus statement provides recommendations for prevention of *C. auris* in pediatric acute care settings, non-acute healthcare settings, and non-healthcare congregate settings. Recommendations incorporate pediatric risk factors and care and address screening practices, isolation precautions, caregiver–infant/child dyad considerations, room placement and rooming in, breastfeeding and skin-to-skin practices, visitation, use of shared spaces, environmental cleaning and disinfection, and management of medical and non-medical equipment, including toys. Recommendations emphasize coordination with local infection prevention and public health partners.

**Conclusions:**

This SHEA consensus statement addresses gaps in pediatric-specific IPC guidance for *C. auris*. The recommendations provide a practical framework to support prevention of transmission within the context of pediatric clinical, developmental, and family-centered care.

## Purpose

Published recommendations for the detection and prevention of *Candida auris* (also referred to as *Candidozyma auris*, see **Terminology**) colonization and infection have primarily focused on adult populations in acute and long-term care healthcare facilities.^1,2^ This document presents consensus-based pediatric recommendations (see Supplementary Material (SM) Table 1) for practical infection prevention and control (IPC) of *C. auris* in acute and non-acute (see Supplementary Material (SM) Table 2) healthcare facilities and non-healthcare congregate settings (see SM Table 2) that care for children, focusing on high-priority gaps in which pediatric and child-serving considerations differ or are lacking from existing guidance.

### Terminology

We use *Candida auris* (*C. auris*) in this document to facilitate clear clinical recognition and communication, while acknowledging that *Candidozyma auris* has been proposed as a taxonomic revision to *Candida auris* and is used in some literature and guidance.

Definitions of populations, settings, practices, precautions, interventions, and other terms relevant to this consensus statement are provided in SM Table 2. This document uses the term Contact Precautions (see SM Table 2) consistent with the Centers for Disease Control and Prevention (CDC) recommendations for *C. auris*. Some facilities may implement additional measures (eg, enhanced environmental disinfection; see SM Table 2) as part of organism-specific precautions. Accordingly, Contact Precautions should be interpreted as inclusive of facility-specific precautions implemented for patients colonized or infected with *C. auris*.

## Sponsorship and intended use

This consensus statement was sponsored by the Society for Healthcare Epidemiology of America (SHEA). It was developed in accordance with the process outlined in the *Handbook for SHEA-Sponsored Guidelines and Expert Guidance Documents, Consensus Statements, and Practice Statements.* ^3^ Consensus statements are developed to address timely topics characterized by limited, evolving, or heterogeneous evidence and therefore cannot account for all clinical circumstances. Accordingly, the recommendations presented here are intended to inform decision-making alongside the judgment of qualified healthcare professionals.

This consensus statement provides IPC recommendations tailored to pediatric populations, recognizing age-specific risk factors, patterns of exposure, and unique aspects of pediatric care, including the role of families. Recommendations are based on a synthesis of identified evidence, theoretical rationale, current practice, practical considerations, expert consensus and assessment of potential harms. The recommendations with remarks are presented in this text and summarized in SM Table 1.

## Methods

Members of the SHEA Pediatric Epidemiology and Antimicrobial Stewardship (PEAS) group identified and prioritized the need to develop pediatric-specific IPC recommendations for *C. auris*. In accordance with the SHEA consensus statement process, PEAS proposed the topic and scope of this consensus statement and identified priority questions related to the IPC of *C. auris*.

The proposed scope, priority questions, and preliminary writing group membership and leadership were reviewed by the SHEA Guidelines Committee and the SHEA Board of Trustees, which provided feedback to refine the proposal. Following revisions in response to this feedback, the proposal was approved, and SHEA convened a multidisciplinary expert panel with representatives from SHEA, the Association for Professionals in Infection Control and Epidemiology (APIC), and the Pediatric Complex Care Association (PCCA), as well as subject-matter experts with relevant experience and expertise (eg, neonatology, early childhood care and education, and long-term care). In accordance with the *SHEA Handbook*,^3^ panelists were selected based on subject matter expertise and experience in pediatric infectious diseases, IPC, and non-acute and non-healthcare settings. The panel included two members who partner with patients and families: Patricia Budo with PCCA and Amanda Green with the Healthy Child Care Initiatives for Child Care Aware of America. The SHEA Guidelines Committee and SHEA Board of Trustees independently reviewed the proposed panel for expertise and perspectives relevant to the scope of the document and provided feedback to refine and finalize the composition. Conflicts of interest were disclosed and managed in accordance with SHEA policy.

### Scope

These recommendations are intended for healthcare personnel (HCP) and caregivers (see SM Table 2) caring for pediatric patients colonized or infected with *C. auris* in acute healthcare settings (eg, hospitals), non-acute healthcare settings (eg, long-term care facilities), and non-healthcare congregate settings (eg, K-12 schools or residential facilities such as Ronald McDonald Houses). See SM Table 2.

This document includes **General Recommendations** (see SM Table 2) derived from existing, published guidance issued by public health authorities and professional societies. These cited recommendations are largely informed by guidance for care of adult populations and provide the foundation for the document’s **Additional Pediatric Recommendations**. Pediatric recommendations address a range of settings and partners, including neonatal intensive care units (NICUs; see SM Table 2), pediatric hospitals and units, including well-baby nurseries, non-acute healthcare facilities, and non-healthcare congregate settings.

Recommendations include screening practices; use of Contact Precautions (see SM Table 2) and personal protective equipment (PPE); room placement and rooming in (see SM Table 2) strategies; breastfeeding and skin-to-skin practices for infants; visitation policies; decolonization (see SM Table 2); management of supplies and medical and non-medical equipment, including toys (see SM Table 2); environmental cleaning (see SM Table 2) and disinfection (see SM Table 2); and educational content for caregivers, visitors (see SM Table 2), and HCP in healthcare and non-healthcare settings.

### Development of recommendations

The panel was divided into four work groups that focused on: (1) screening for *C. auris*, (2) patients or caregivers (see SM Table 2) who are colonized (see SM Table 2) or infected (see SM Table 2) with *C. auris*, (3) management of shared equipment or environment, and (4) non-acute healthcare settings and congregate non-healthcare settings (see SM Table 2).

Work groups conducted a structured, non-systematic review of relevant peer-reviewed literature and an environmental scan with summaries of relevant gray literature (including guidance from governmental agencies, professional societies, and state, local, and international public health authorities). When applicable, IPC guidance for other multidrug-resistant organisms (eg, methicillin-resistant *Staphylococcus aureus*) was reviewed to inform specific considerations. Evidence from these sources was integrated with expert judgment to draft proposed recommendations and accompanying remarks.

Recommendations were refined through an iterative consensus process that included discussion during virtual meetings and communication among work groups and co-chairs.

### Voting and approval

Each pediatric recommendation underwent anonymous electronic voting using predefined thresholds to categorize outcomes as unanimous consensus (100% agreement), consensus (≥80% to <100% agreement), majority opinion (51% to <80% agreement), or no recommendation (≤50% agreement).^3^ Twenty panelists participated in live voting. Three additional panelists who were unable to attend the live session contributed to earlier rounds of voting and were given the opportunity to raise concerns prior to finalization. Voting results, including the level of agreement, are reported for each recommendation. Panelists with relevant conflicts of interest recused themselves from discussion and voting on affected recommendations in accordance with SHEA policy.^3^ Panelists could also abstain from voting when they thought they lacked sufficient expertise or information to evaluate a specific recommendation.

Preambles and remarks accompanying the recommendations provide additional context, clarification, or implementation considerations. These sections were not subject to separate voting but were reviewed and approved by the panel as part of the final document approval process.

The final document underwent review by the SHEA Guidelines Committee, APIC, the American Society for Microbiology (ASM), PCCA, and Ronald McDonald House Global. It was endorsed by the SHEA Board of Trustees, APIC, ASM, the Pediatric Infectious Diseases Society (PIDS), PCCA, and Ronald McDonald House Global. Additional organizations that endorsed this document after publication are listed on the SHEA website.

## Background

*C. auris* is a multidrug-resistant fungal pathogen causing healthcare-associated infections and outbreaks worldwide with substantial morbidity and mortality.^2,4–7^ *C. auris* can colonize the nares, inguinal folds, perianal skin, axilla, hands, or toe webs, often with a high bioburden and is unresponsive to decolonization.^8,9^ *C. auris* can survive for several weeks as biofilms on multiple surfaces, including steel and plastic, and is resistant to many disinfectants.^10^ Despite disinfection with agents effective against *C. auris*, in the presence of a colonized or infected patient, recontamination of surfaces can occur within four hours.^11^ Routes of *C. auris* transmission include indirect contact with the contaminated healthcare environment or contaminated healthcare equipment or direct contact with a colonized or infected individual.^2^

CDC, state health departments, and international organizations provide IPC guidance to mitigate transmission of *C. auris* in healthcare facilities, but there is little guidance specific to pediatric populations.^1,12–14^ Emerging literature describes *C. auris* infections and outbreaks in pediatric healthcare facilities and NICUs worldwide and underscores the importance of early detection and reporting, isolation, and rigorous environmental disinfection to reduce transmission as advocated for adult populations.^15–18^ However, as settings and patterns of pediatric healthcare vary from those of adults, pediatric-specific IPC strategies for *C. auris* are needed. For example, in pediatrics, a caregiver is typically present at the bedside in extended close contact with their child; thus, IPC risk mitigation and education should include the caregiver-patient dyad.^19–22^ Children may be in facilities with shared (see SM Table 2) diagnostic and therapeutic areas, NICUs configured with multiple isolettes in a single, large space, shared playrooms and lounges, and shared breastfeeding rooms. Intimate contact between caregivers and infants through breastfeeding and skin-to-skin practices (see SM Table 2) are universally endorsed for their health benefits to the dyad.^23–26^ Unique care patterns also exist in non-healthcare congregate settings for children which have communal spaces and may have overnight accommodations for children and families during medical treatments and recovery.^27^ As *C. auris* remains relatively rare in pediatrics, successful implementation and adherence to IPC recommendations require coordinated communication among healthcare facilities, non-healthcare congregate settings, public health partners (see SM Table 2), and caregivers and families provided in plain language (see SM Table 2).

### Risk factors

Certain epidemiologic exposures and clinical risk factors can increase adult and pediatric patients’ risk of *C. auris* colonization or infection. Relevant epidemiologic factors include frequent or prolonged inpatient hospitalizations, particularly when sharing a room, care area, or mobile equipment with another patient colonized or infected with *C. auris*, receipt of care in a healthcare facility with suspected, confirmed, or ongoing *C. auris* transmission, or residence in a community with high *C. auris* transmission rates.^15,28–30^ Potential additional epidemiologic risk factors specific to pediatric patients may include having a birth parent or caregiver colonized or infected with *C. auris* and being breastfed by a caregiver colonized with *C. auris.* ^31^

Clinical risk factors across age groups include the presence of indwelling medical devices (eg, central venous catheters (CVCs), feeding tubes, or urinary catheters), mechanical ventilation, receipt of parenteral nutrition, and exposure to broad spectrum antimicrobials, including third-generation cephalosporins or carbapenems, or prolonged antifungal prophylaxis.^15,29,32^ Additional factors described in pediatric populations include extreme prematurity, congenital heart disease, malnutrition, and sharing space, equipment, or diagnostic areas with patients, including adult patients, colonized or infected with *C. auris.* ^15–18,32–37^

## Consensus recommendations for prevention of *C. auris* in pediatrics

### Acute healthcare facilities

#### Public health and facility response

Given the relative rarity of *C. auris* in the pediatric population, collaboration and communication with local IPC (see SM Table 2) and public health partners are essential.^14,30,39^

**General recommendations**

- Report possible or confirmed cases of *C. auris* infection immediately to the clinical team, local IPC, and public health partners. Reporting thresholds may vary by jurisdiction resulting in different recommendations in certain circumstances^7,14,39,40^; however, healthcare facilities should have a low threshold for reporting cases in pediatric patients to public health partners.
- Collaborate with local IPC and public health partners to receive guidance and determine the response for newly identified cases of *C. auris*, suspected or confirmed transmission of *C. auris* in a healthcare facility, or high rates of local community transmission.^14,30,39^

**No additional pediatric recommendations.**

#### Identifying patient risk factors and risk mitigation

**General recommendations**

- Identify the patient’s risk factors for *C. auris* colonization or infection which may include^15,28,30,41^:
  - Known epidemiologic link to *C. auris* and/or clinical risk factors:
    - Exposure in a healthcare facility, eg, sharing the same room, spending time in a shared care area with a patient with *C. auris* even if the person with *C. auris* has been discharged or is no longer in the care area, or using mobile equipment that was used by a patient with *C. auris* colonization or infection.
    - Care in a healthcare facility with current, suspected, or confirmed *C. auris* transmission or in a community with high *C. auris* transmission rates.
    - Whenever a patient who is colonized or infected with *C. auris* is transferred to another facility, include their *C. auris* status in the medical record and in verbal and written transfer information. Also provide whether the facility has current, suspected, or confirmed *C. auris* transmission.
  - Clinical factors:
    - Presence of an indwelling medical device (eg, CVC, tracheostomy tube, gastrostomy tube, urinary catheter), mechanical ventilation, or the use of parenteral nutrition.
    - Treatment with broad spectrum antimicrobials such as third generation cephalosporins or carbapenem agents or prolonged antifungal prophylaxis.
- Risk mitigation:
  - Assess the need for all modifiable risk factors daily, including use of indwelling medical device(s) and antimicrobials.^41–45^
  - Adhere to care bundles for medical device care.^42–44^

**No additional pediatric recommendations.** See **Background, Risk Factors section** for risk factors unique to pediatric populations.

#### Education, training, monitoring, and adherence

At the time of developing these recommendations, *C. auris* infection or colonization remains relatively rare in pediatrics. HCP, caregivers, and other close contacts (see SM Table 2), for example household members, may be unaware of this pathogen and unfamiliar with strategies to prevent transmission in pediatric healthcare and non-healthcare settings.^1^

This section applies to the following circumstances:

- Caregivers or HCP who interact with pediatric patients who are colonized or infected with *C. auris*
- Caregivers who are known to be colonized or infected with *C. auris*
- Caregivers who are at higher risk of *C. auris* colonization or infection due to a known exposure to *C. auris* (see **Risk Factors**) and their underlying health status
- HCP and support staff working in healthcare settings with suspected, confirmed, or ongoing *C. auris* transmission

**General recommendations**

- Provide HCP and support staff in facilities with suspected, confirmed, or ongoing *C. auris* transmission with regular education and training on prevention of *C. auris.* ^46^ Include the following^1,2,47^:
  - Risks of environmental transmission of *C. auris*
  - Proper cleaning and disinfection protocols
  - IPC protocols
  - When to suspect *C. auris* based on epidemiologic risk factors and clinical context and when to notify local IPC or infectious diseases specialists.
- Monitor adherence to IPC protocols and provide feedback to staff and leadership.^2,13^

**Additional pediatric recommendations**

**1.** *(unanimous consensus)* Educate the caregivers, family members, and visitors of a child in an acute care setting who is infected or colonized with *C. auris* about strategies to reduce the risk of acquisition or transmission of *C. auris* during hospitalization.^2,13^ Include the following education:

- Components of Standard Precautions (see SM Table 2)
- When and how to perform hand hygiene
- When and how to safely don and doff PPE, especially gloves and gowns.

Remarks:

- As children require close contact with their caregivers for their care and well-being, provide verbal and written education in plain language describing prevention strategies for *C. auris* during hospitalization and at hospital discharge to mitigate the risk of *C. auris* transmission.^13^
- Caregivers and visitors should be educated to avoid shared spaces and contact with other patients, and their movement within the facility should be limited to direct room-to-exit routes.^1^
- Educate visitors to always wear PPE for Contact Precautions (ie, gloves and gowns) while in the patient room.^2,13^ This does not apply to caregivers who are rooming in with patients (see **Recommendation 8**).
- Reinforce education during prolonged hospitalizations (*expert opinion*).

**2.** *(unanimous consensus)* Educate caregivers and visitors who are known to be colonized or infected with *C. auris* about strategies to reduce transmission to the hospitalized pediatric patient and reduce transmission to close contacts after discharge (see **Recommendation 1** for topics of education).^2,13,48^

Remark:

- If a caregiver or visitor is known to be colonized or infected with *C. auris*, document this in the child’s medical record.

**3.** *(consensus)* Provide caregivers with discharge information on *C. auris* in plain language that includes disclosure information (see **Recommendations 34 and 35**) and strategies to prevent transmission of *C. auris* in healthcare and non-healthcare congregate settings.^2,27,49,50^

Remarks:

- The information may be shared with HCP in ambulatory care and non-healthcare congregate settings.
- Consensus achieved with 95% agreement (19 of 20 panelists agreed, 0 disagreed and 1 abstained).

#### Surveillance and screening

Currently, approaches to surveillance and screening for *C. auris* vary across settings and epidemiologic context and are not standardized.^51^ Screening may be used to determine if exposed patients (see SM Table 2) have become colonized.

**General recommendations**

- Place exposed patients who have a positive screening test for *C. auris* on Contact Precautions.
- When a facility suspects or identifies ongoing *C. auris* transmission, perform point prevalence surveys every 7 to 14 days on the entire unit, not just sub-sections of the unit.^18,39,52^
  - Reduce the frequency of the surveys as transmission decreases.^39^

- Perform contact tracing of additional patients (not on the unit) at high risk for *C. auris* and include in point prevalence surveys (see **Risk Factors**).^14,15,28,39,41,53^
- Do not re-screen patients with known *C. auris* colonization or infection to assess their status or to remove Contact Precautions.^41^
- Do not screen healthcare personnel (HCP) for *C. auris.* ^41^

**Additional pediatric recommendations**

**4.** *(unanimous consensus)* Do not screen patients admitted to the NICU or other acute units unless one of following known exposures to *C. auris* exists (*expert consensus*):

- A birth parent, caregiver, or other close contact is colonized or infected with *C. auris.*
- The patient is breastfed by a caregiver who is colonized or infected with *C. auris.*
- The patient is sharing space or equipment with patients, including adult patients, who have *C. auris* colonization or infection.
- The patient was transferred from a facility with suspected, confirmed, or ongoing *C. auris* transmission.

Remarks:

- Undertake screening in coordination with local IPC and public health partners.
- For healthy newborns who (1) are rooming in (see SM Table 2) with a birth parent colonized or infected with *C. auris* and (2) do not require NICU care, routine screening is not recommended because screening results would not alter immediate management (see **Recommendation 10**).
- Do not perform screening solely on the basis of transfer from another healthcare facility, including acute care hospitals, institutions with mixed adult and pediatric patients, long-term care or rehabilitation facilities, or facilities outside of the United States, unless there is suspected, confirmed, or ongoing *C. auris* transmission in the transferring facility.^14,15,28,39,41,53^ The risk of interfacility transmission is lower for pediatric patients because: *C. auris* is rare in pediatric facilities; pediatric long-term care and postacute care facilities have fewer transfers to acute care hospitals; and pediatric facilities are sparsely distributed and patient volumes are lower.^53,54^
- If pediatric patients share spaces (eg, radiology suite, surgical suite, procedure room) or equipment with other patients, including adult patients, with *C. auris* colonization or infection or with adult or pediatric patients linked to *C. auris* transmission, consult local IPC partners to determine which pediatric patients should be screened.^14,15,28,39,41,53^
- Identified evidence was insufficient to inform the timing and frequency for screening children for *C. auris* who have ongoing, prolonged exposure to a caregiver or other close contact who is colonized or infected with *C. auris*.

**5.** *(consensus)* For an infant in the NICU whose birth parent is colonized or infected with *C. auris*, screen the infant to inform IPC decisions and empiric antimicrobial therapy, using the following recommended screening intervals^31,55,56^:

- At birth, after bathing
- On the seventh day of life if the neonate (see SM Table 2) remains hospitalized
- Every 2 weeks thereafter if the infant remains hospitalized
- As needed to inform decision-making
- At hospital discharge
- At readmission.

Remarks:

- Identified evidence was insufficient to determine how long an infant who was discharged from the NICU remains at risk of colonization or infection.
- An 800-gram, 25-week neonate acquired *C. auris* within hours of birth following vaginal delivery to a birth parent with a positive vaginal swab.^31^
- In patients with recent admission to an adult ICU setting with ongoing *C. auris* transmission, the median interval between the last negative and first positive screening sample was 6 days (IQR 4–12).^55^
- In an adult trauma ICU outbreak, all patients (n = 10) became colonized with *C. auris* by hospital day 4.^56^
- Consensus achieved with 85% agreement (17 of 20 panelists agreed, 0 disagreed and 3 abstained).

**6.** *(consensus)* Do not screen caregivers and other close contacts of pediatric patients colonized or infected with *C. auris* unless the caregiver or other close contact requires frequent inpatient healthcare interactions.^14,39,41,57^

Remarks:

- Advise caregivers and other close contacts with frequent inpatient healthcare hospitalizations to discuss being screened for *C. auris* with the healthcare team managing their inpatient care. Screening results can inform whether Contact Precautions are needed for their subsequent admissions and/or empiric antimicrobial treatment.^2,39,57^
- Identified evidence was insufficient to inform the timing and frequency of screening high-risk caregivers and other close contacts expected to have repeated, long-term exposure to their child, including with bodily fluids and skin.
- Consensus achieved with 85% agreement (17 of 20 panelists agreed, 2 disagreed and 1 abstained).

##### Swab collection

**General recommendations**

- Obtain a composite swab of the patient’s bilateral axilla and groin.^58^

**Additional pediatric recommendations**

**7.** *(majority opinion)* Obtain a bilateral composite swab of the patient’s nares, axilla, and groin.^11,41,56,59^

Remarks:

- Identified evidence was insufficient regarding the relative yield of different body sites for *C. auris* screening.
- During a NICU outbreak, investigators assessed composite skin swabs of the axilla and groin and identified *C. auris* by PCR in 15% (29/195) of samples.^18^
- In adults, the addition of nares or hands to axilla and groin swabs increased the recovery of *C. auris.* ^11,59^
- In nursing home patients, swabs of anterior nares, external auditory canal, axilla, inguinal crease, perianal skin, toe webs, palm and fingertips were performed, and patterns of body-site colonization were variable.^9^
- Other studies included the ears, eyes, and oral sites, including a composite swab of a neonate’s axilla, groin, eye, and ear.^31,56^
- If resources permit, *C. auris* screening may also include swabbing the patient’s hands.^11,59^
- When using a single composite swab in accordance with laboratory instructions, collect specimens from clean to dirty sites (nares, then axilla, then groin) (*expert opinion*).
- If collecting specimens separately with different swabs (eg, one swab for the nares and one for the axilla and groin), the swabs may be combined in a single transport medium, if approved by the local laboratory or submitted as separate specimens.
- Majority opinion with 75% agreement (15 of 20 panelists agreed, 2 disagreed and 3 abstained).

##### Cultures and PCR

**General recommendations**

- Depending on local resources, real-time polymerase chain reaction (PCR) is the preferred method for detecting colonization but may detect non-viable organisms.^60,61^
- If performing culture, use a *Candida* chromogenic medium and incubate at the temperature stated in the manufacturers’ instructions for use (MIFU) to recover isolates for species confirmation.^60^
- Current Matrix-Assisted Laser Desorption/Ionization Time-of-Flight Mass Spectrometry (MALDI-TOF MS) systems can reliably identify isolates of *C. auris*. Variable performance has been reported for biochemical test systems.^61^
- Depending on local resources, facilities may process swab specimens for *C. auris* culture with or without broth enrichment or perform PCR directly from swab specimens.^60^
- Local IPC in facilities without the capacity to perform *C. auris* screening should coordinate with public health partners to perform screening testing in a reference laboratory or a public health laboratory. The CDC Antimicrobial Resistance Laboratory Network offers free PCR-based colonization testing for healthcare facilities and health departments.^41^

**No additional pediatric recommendations.**

#### Contact precautions and hand hygiene

**General recommendations**

- Implement Contact and Standard Precautions for patients infected or colonized with *C. auris.* ^2^
- Ensure HCP, caregivers, and visitors perform hand hygiene before entering and after exiting the room of a patient infected or colonized with *C. auris* and before and after direct contact with a patient or their environment or items.^2^
- Perform hand hygiene using alcohol-based hand sanitizer or soap and water when hands are visibly soiled.^2,39,62,63^
- Gloves are not a substitute for hand hygiene.^2,62,63^ Perform hand hygiene before putting on gloves and after removing gloves.
- Maintain Contact Precautions for the duration of hospitalization and during future acute care admissions.^2,41^

**Additional pediatric recommendations:** **Infants and children** **colonized or infected with *C. auris***

**8.** *(consensus)* Implement Contact and Standard Precautions for *C. auris* per local IPC and public health recommendations.

Remarks:

- For pediatric patients with prolonged hospitalizations who experience mental health decline attributable to environmental monotony, collaborate with local IPC partners to assess whether children can safely leave their hospital room for reasons other than a medical procedure (expert opinion).
- Caregivers are not expected to wear PPE continuously while rooming in. Caregivers should follow facility policies for PPE outside the patient’s room (see **Room placement and cohorting**).
- Consensus achieved with 95% agreement (19 of 20 panelists agreed, 1 disagreed and 0 abstained).

**9.** *(consensus)* Work with local IPC and public health partners to determine the duration of Contact Precautions for a pediatric patient’s current and future admissions to acute care (*expert opinion*).

Remarks:

- Identified evidence was insufficient to inform the duration of Contact Precautions for *C. auris* for infants and children for current and future admissions.
- Contact precautions for all future admissions may impact the clinical care of infants or children with complex medical needs who will have frequent healthcare interactions over many years or may cause anxiety and stress in such children and/or their caregivers.^64,65^
- Consensus achieved with 90% agreement (18 of 20 panelists agreed, 2 disagreed and 0 abstained).

**Additional pediatric recommendations:** **Caregivers** **colonized or infected with *C. auris***

**10.** *(consensus)* Place hospitalized newborns, infants (including those in the NICU), and children on Contact Precautions if their caregiver is known to be colonized or infected with *C. auris* (*expert opinion*).

Remarks:

- When a rooming in caregiver is colonized with *C. auris* and the child is in a single room, the caregiver does not need to wear PPE while in the patient’s room. The patient should remain on Contact Precautions so that HCP will continue to implement all required components.
- Caregivers should avoid shared spaces and contact with other patients, and movement within the facility should be limited to direct room-to-exit routes.^1^
- Consensus achieved with 90% agreement (18 of 20 panelists agreed, 0 disagreed and 2 abstained).

**11.** *(consensus)* If known, document the caregiver’s colonization or infection with *C. auris* in the patient’s medical record for implementation of Contact Precautions for *C. auris* for the patient in the case of future healthcare admissions, including non-acute healthcare facilities (*expert opinion*).

Remarks:

- Identified evidence was insufficient to inform the use of Contact Precautions for *C. auris* for future admissions if the child’s screening cultures are negative. However, the risk remains for transmission of *C. auris* to the child from continued close contact with their caregiver or other close contacts colonized or infected with *C. auris*.
- Consensus achieved with 85% agreement (17 of 20 panelists agreed, 0 disagreed and 3 abstained).

**12.** *(consensus)* For current and future healthcare admissions, including long-term care, consult local IPC and public health partners to determine the appropriate duration for Contact Precautions for the child of a caregiver who is colonized or infected with *C. auris* (*expert opinion*).

Remarks:

- Identified evidence was insufficient to inform the duration of Contact Precautions for infants and children with a caregiver who is colonized or infected with *C. auris*.
- Consensus achieved with 90% agreement (18 of 20 panelists agreed, 0 disagreed and 2 abstained).

#### Room placement and cohorting

**General recommendations**

- Place a patient who has *C. auris* colonization or infection in a single-patient room with a dedicated bathroom, whenever possible.^2^
- If single rooms are unavailable, cohort patients together who have *C. auris* colonization or infection.^2,18^

**Additional pediatric recommendations:** **Infants and children** **colonized or infected with *C. auris***

**13.** *(consensus)* Allow caregivers to room-in with pediatric patients colonized or infected with *C. auris.* ^19,66^

Remarks:

- For overnight stays or extended visits, continuous use of PPE by caregivers may be impractical.^66^ Caregivers who are rooming in are not expected to wear PPE continuously while in the patient’s room, regardless of whether the caregiver or child is colonized or infected with *C. auris*; however, caregivers should follow facility policies for use of PPE outside the patient’s room (see **Recommendation 8**).
- Consensus achieved with 95% agreement (19 of 20 panelists agreed, 1 disagreed and 0 abstained).

**14.** *(consensus)* For NICUs with open bays or pods, follow local IPC and public health partner guidance for placement of isolettes or cribs of infants with *C. auris* colonization or infection (*expert opinion*).

Remarks:

- The distance between isolettes or cribs recommended by local IPC and public health partners may vary depending on local resources.
- The American Academy of Pediatrics and Facilities Guidelines Institute recommend 8 feet of distance between isolettes to facilitate care, privacy, and family involvement and to reduce the risk of transmission of potential pathogens.^67,68^
- Consensus achieved with 95% agreement (19 of 20 panelists agreed, 1 disagreed and 0 abstained).

**Additional pediatric recommendations:** **Caregivers** **colonized or infected with *C. auris***

**15.** *(unanimous consensus)* Allow caregivers who are colonized with *C. auris* to room-in with their child.^19,66^

**16.** *(consensus)* In accordance with local IPC guidance, before a caregiver with active *C. auris* infection rooms-in with a pediatric patient, confirm that they have received appropriate antifungal treatment and are showing signs of improvement.^69^

Remarks:

- Compassionate care visitation laws vary among US states.
- Consensus achieved with 95% agreement (19 of 20 panelists agreed, 0 disagreed and 1 abstained).

#### Breastfeeding and skin-to-skin practices

**No general recommendations.**

**Pediatric recommendations:** **Infants and children** **colonized or infected with *C. auris***

**17.** *(unanimous consensus)* Encourage and facilitate breastfeeding and skin-to-skin practices.^23–26,70^

Remark:

- Identified evidence was insufficient to inform the risk of *C. auris* transmission from infants with *C. auris* colonization or infection to parents or caregivers during breastfeeding or skin-to-skin practices.

**18.** *(unanimous consensus)* Use shared decision-making (see SM Table 2) to guide breastfeeding and skin-to-skin practices for caregivers with underlying medical conditions or frequent inpatient healthcare exposures (*expert opinion*).

Remark:

- Extrapolating from other guidance, if expressed breast milk (EBM) is stored, double-bag EBM and place in a separate freezer that is cleaned and disinfected daily. If a separate freezer is not available, place EBM on a dedicated freezer shelf that is cleaned and disinfected daily.^71^

**Pediatric recommendations:** **Caregivers** **colonized or infected with *C. auris***

**19.** *(consensus)* Use shared decision-making to guide breastfeeding and skin-to-skin practices for caregivers who are colonized or infected with *C. auris*, including decisions about cleansing the caregiver’s breasts or skin with soap and water before breastfeeding or skin-to-skin practices (*expert opinion*).

Remarks:

- Identified evidence was insufficient to inform the risk of transmission of *C. auris* from the breast milk of caregivers colonized or infected with *C. auris*.
- Breast milk does not need to be tested for *C. auris* before feeding the infant.
- The immune-protective benefits of breastfeeding are well described.^23,25,26^
- Identified evidence was insufficient to inform the benefits or harms of breast cleansing before breastfeeding or skin cleansing before skin-to-skin contact by caregivers who are colonized or infected with *C. auris*.
- Cleansing with soap and water may remove the caregiver’s normal skin flora which could interfere with the transfer of beneficial flora to preterm infants during skin-to-skin contact. This transfer of normal flora supports immune development, reduces colonization with potential pathogens, and accelerates oral microbiome maturation.^24,72–74^
- If EBM is stored, double-bag EBM and place in a separate freezer that is cleaned and disinfected daily or if a separate freezer is not available, place EBM on a dedicated freezer shelf that is cleaned and disinfected daily.
- Consensus achieved with 95% agreement (19 of 20 panelists agreed, 0 disagreed and 1 abstained).

**20.** *(consensus)* Instruct a caregiver with *C. auris* infection involving the milk ducts or an active cutaneous lesion involving the breast to avoid breastfeeding or to discard EBM (*expert opinion*).

Remark:

- Consensus achieved with 90% agreement (18 of 20 panelists agreed, 0 disagreed and 2 abstained).

**21.** *(consensus)* Instruct a caregiver with *C. auris* infection to prevent the infant from contacting areas with active infection (*expert opinion*).

Remark:

- Consensus achieved with 95% agreement (19 of 20 panelists agreed, 0 disagreed and 1 abstained).

#### Visitation and common spaces

**No general recommendations**

**Pediatric recommendations**

**22.** *(consensus)* Consult local IPC to develop policies for pediatric patients with *C. auris* colonization or infection, including patients in the NICU or the newborn nursery, that address the following (*expert opinion*):

- Visitation by siblings
- Visitation by caregivers or visitors who are colonized or infected with *C. auris*
- PPE use by caregivers who are rooming in
- PPE use by visitors who are not rooming in
- Temporary transport to shared care areas (eg, procedural areas)
- Appropriate use of common spaces (eg, kitchens, washing machines, lounges, child life areas) by caregivers, visitors, and siblings, including any necessary restrictions or mitigation strategies.

Remarks:

- For overnight stays or extended visits, continuous use of PPE by caregivers may be impractical.^66^ (see **Recommendations 8, 10, 13**).
- Extrapolating from other guidance, crowded patient rooms, bedsides of multi-patient rooms, or NICU pods increase the risk of transmission and the number of potentially exposed individuals.^70,75^
- Consider risks to visitors with underlying medical conditions.^19,66^ (see **Recommendation 6**)
- Use shared decision-making with caregivers to determine appropriate visitation.
- In accordance with guidance from local IPC and public health partners, visitation may be temporarily restricted during *C. auris* outbreaks.^66^
- Discuss exceptions for compassionate care visitation. Compassionate care laws vary by US state.
- Consensus achieved with 95% agreement (19 of 20 panelists agreed, 1 disagreed and 0 abstained).

#### Decolonization

**General recommendations**

- Currently, no agent has been identified to be effective and efforts to decolonize do not prevent transmission of *C. auris.* ^2,14^
- Emphasize personal hygiene, especially of known colonized skin areas (such as the axillae, inguinal folds, palms) which can decrease the bioburden of *C. auris* and therefore reduce the likelihood of transmission.^16^
- The effects of chlorhexidine on reducing skin burden or infection with *C. auris* have not been systematically studied.^1,2^

**No additional pediatric recommendations.**

#### Supplies, medical and non-medical equipment, and environmental cleaning and disinfection

**General recommendations**

- For patients with *C. auris* colonization or infection^1,2,39^
  - Use single-patient or disposable equipment whenever possible.
  - Use single-patient supplies, such as sterile ultrasound gel packets.
  - Use impermeable, sealed bags to handle linens and waste.
  - Use standard healthcare laundering.
- At least daily, clean and disinfect the rooms of patients with *C. auris* colonization or infection with an EPA-registered product effective against *C. auris.* ^2,29,76^
- Terminally clean (see SM Table 2) the patient’s room at hospital discharge and shared spaces (eg, occupational and physical therapy areas) that were used by patients with *C. auris* colonization or infection.^2^
- Clean and disinfect medical equipment after each use by patients with *C. auris* colonization or infection (eg, glucometers, ventilators, ultrasound machines, blood pressure cuffs).^2,12^
- Label disinfected medical equipment and separate it from dirty equipment.^2^
- For disinfection of rooms and mobile and reusable equipment, use EPA-registered products (see SM Table 2) effective against *C. auris* (List P).^2,14,77^
- Follow the MIFU for disinfectants’ contact times.^77^
- Data on “no touch” devices such as germicidal UV irradiation and vaporized hydrogen peroxide are limited and parameters for effective disinfection are not well understood. Facilities may use these supplemental methods only after completing recommended standard cleaning and disinfection.^2^

**Additional pediatric recommendations**

**23.** *(consensus)* Follow disinfection requirements for specialized pediatric equipment, including items with complex MIFUs, such as incubator and warmer beds, isolettes, milk warmers, X-ray plates and machines, ultrasound probes and machines, ophthalmology equipment, and audiology equipment.^1,67^

Remarks:

- If possible, dedicate equipment for physical and occupational therapy to a single patient and perform sessions in the patient’s room.
- Consensus achieved with 90% agreement (18 of 20 panelists agreed, 1 disagreed and 1 abstained).

**24.** *(consensus)* Select non-medical equipment (eg, toys, tablets, video game consoles, and controllers) that are compatible with an EPA-registered product effective against *C. auris* and establish cleaning and disinfecting protocols with local IPC and child life specialists (*expert opinion*).

Remarks:

- Preferentially choose non-porous toys that can be cleaned and disinfected at discharge or porous toys that remain with the patient throughout hospitalization.^78^ Options for processing toys at patient discharge include:
  - Placing toys in a plastic bag and instructing families to wash the toys at home in hot water.
  - Cleaning and disinfecting non-porous toys for re-use.
  - Discarding the toys if unable to effectively clean and disinfect.
- Consensus achieved with 90% agreement (18 of 20 panelists agreed, 0 disagreed and 2 abstained).

**25.** *(consensus)* If possible, dedicate toys for exclusive use by an infant or child on Contact Precautions for *C. auris.* ^78^

Remark:

- Consensus achieved with 90% agreement (18 of 20 panelists agreed, 0 disagreed and 2 abstained).

**26.** *(consensus)* For caregivers colonized or infected with *C. auris*, and for infants colonized or infected with *C. auris*, dedicate breast pumps and accessories for exclusive use by the breastfeeding caregiver to use in the infant’s room. Clean and disinfect the pump and accessories after each use with an EPA-registered product effective against *C. auris.* ^77,79^

- If a single patient room is not available, use an individual lactation room. Clean and disinfect high-touch surfaces (see SM Table 2) and equipment after each use with an EPA-registered product effective against *C. auris.* ^77,80,81^
- If an individual lactation room is not available, use the communal lactation room. Clean and disinfect high-touch surfaces and equipment after each use with an EPA-registered product effective against *C. auris.* ^2,79^

Remark:

- Consensus achieved with 85% agreement (17 of 20 panelists agreed, 1 disagreed and 2 abstained).

**27.** *(consensus)* For pediatric patients whose caregiver is *C. auris* colonized or infected, clean and disinfect the patient’s room and medical and non-medical equipment (see **General Recommendations**, **Recommendation 23**, **Recommendation 26**) at least daily (*expert opinion*).

Remarks:

- The patient’s room and equipment have likely become contaminated with *C. auris* from their caregiver, which poses ongoing risk to the child and other patients on the unit.
- Consensus achieved with 90% agreement (18 of 20 panelists agreed, 1 disagreed and 1 abstained).

#### Communication and documentation

**General recommendations**

- Place alerts in the medical record that indicate patients with *C. auris* colonization or infection.^2^
- Place visible signage for Contact Precautions outside the patient’s room.^2^
- Limit transport of patients with *C. auris* colonization or infection to medically necessary procedures.^2^
- To ensure continuity of Contact Precautions, notify receiving departments or facilities of a patient’s *C. auris* status.^2^

**No additional pediatric recommendations.**

### Non-acute healthcare settings

Long-term care and rehabilitation facilities for children are unique non-acute healthcare settings. Pediatric residents often consider these settings their “home,” caregivers and visitors actively participate in the long-term care of their children residing in these settings, and non-acute healthcare facilities have different designs than acute care facilities. Shared patient rooms are common. Other shared spaces, crucial to therapeutic interventions and quality of life, are the norm and include on-site classrooms for K-12 education or early childhood education, play areas with shared toys or equipment, dining and kitchen areas, diapering stations, therapy spaces, and pools. HCP in non-acute healthcare settings also engage with acute care facilities, public health partners (per local regulations), childcare resources and referrals, and the department of education.

The risks of transmission and acquisition of *C. auris* are likely based on the different care needs of residents. During activities that do not involve direct contact with secretions or excretions, for example attending school, the risks are likely low. During activities that involve limited or controlled contact with secretions or excretions, for example diaper changes without diarrhea, assisting with eating, or localized wound care, the risks are likely moderate. During activities with extended contact with secretions or excretions, for example when manipulating devices, bathing, or diaper changes for diarrhea, the risks are likely higher.

**General recommendations**

Comprehensive recommendations for nursing homes caring for adult patient populations have been published.^82^

**Pediatric recommendations**

**28.** *(consensus)* Do not exclude infants and children with *C. auris* colonization or infection from pediatric non-acute healthcare settings.^83^

Remarks:

- Consistent with other transmissible pathogens, children with uncontrolled drainage with *C. auris* cannot participate in group activities.^16,27,83^
- Consensus achieved with 95% agreement (19 of 20 panelists agreed, 0 disagreed and 1 abstained).

**29.** *(consensus)* Consult local IPC and public health partners to determine the duration of Contact Precautions for a pediatric resident’s current and future admissions to non-acute care facilities (*expert opinion*).

Remarks:

- Consensus achieved with 90% agreement (18 of 20 panelists agreed, 2 disagreed and 0 abstained).

**30.** *(consensus)* Use Standard Precautions with an emphasis on hand hygiene when caring for residents with *C. auris* colonization during low or moderate-risk activities involving controlled or minimal contact with secretions or excretions (eg, school interactions, diaper changes without diarrhea, assistance with eating, or localized wound care) (*expert opinion*).

Remarks:

- While high rates of *C. auris* transmission and colonization have been described in nursing homes caring for adults,^76,84^ identified evidence was insufficient to characterize the risk of *C. auris* transmission or colonization in pediatric long-term care and rehabilitation facilities.
- In a point prevalence survey of *C. auris* colonization in a pediatric long-term transitional care facility, no residents (n = 29) tested positive.^53^
- Consensus achieved with 85% agreement (17 of 20 panelists agreed, 2 disagreed and 1 abstained).

**31.** *(consensus)* Use Enhanced Barrier Precautions (gloves and gowns; see SM Table 2) when caring for residents during higher risk activities that involve extended contact with secretions or excretions (eg, manipulating medical devices, bathing, or changing the diaper of a resident with diarrhea).^27,85^

Remarks:

- In adult non-acute healthcare settings, contact with the contaminated environment, contaminated patient-care equipment, or ineffective hand hygiene by HCP has led to transmission of *C. auris.* ^28^
- Identified evidence was insufficient to determine the risk of direct patient-to-patient transmission of *C. auris* in pediatric non-acute healthcare settings. The risk has not been studied in pediatric long-term care.
- Continual or indiscriminate use of Enhanced Barrier Precautions,^86^ as advocated for adult nursing home residents, may negatively affect pediatric residents’ cognitive and behavioral development and participation in therapies.
- Consensus achieved with 90% agreement (18 of 20 panelists agreed, 1 disagreed and 1 abstained).

**32.** *(consensus)* Consult local IPC and public health partners, when relevant, to develop visitation policies for: (*expert opinion*)

- Pediatric residents who are colonized or infected with *C. auris*
- Sibling visitation for pediatric residents who are colonized or infected with *C. auris*
- Caregivers or visitors who are colonized or infected with *C. auris*.

Remarks:

- Limit the number of visitors in the residents’ rooms, or at the bedside for multi-resident rooms. In adult nursing homes, crowded nursing homes with a high proportion of shared bedrooms and bathrooms increase the risk of transmission of respiratory viral infections and the number of potentially exposed individuals.^87^
- Use shared decision-making with caregivers to decide who may visit the pediatric resident who is colonized or infected with *C. auris*.
- Consider risks to visitors with medical conditions.^19^
- Seek guidance from local IPC and public health partners to temporarily restrict visitation during *C. auris* outbreaks.^66^
- Consensus achieved with 95% agreement (19 of 20 panelists agreed, 0 disagreed and 1 abstained).

### Non-healthcare congregate settings

See SM Table 2 for definitions of non-healthcare congregate settings. Examples of HCP who work in non-healthcare congregate settings include school nurses, nurses at pediatric or family residential facilities, and nurses providing care to individual children.

**No general recommendations.**

**Pediatric recommendations**

**33.** *(unanimous consensus)* Do not exclude children colonized with *C. auris* from non-healthcare congregate settings.^83^

Remarks:

- Identified evidence was insufficient to determine the risk of transmission of *C. auris* in non-healthcare congregate settings for children.
- Extrapolating from other guidance, children with draining wounds with *C. auris* should be excluded from early childcare until drainage is contained.^88,89^
- Extrapolating from other guidance, children should not attend school if they have an open draining wound that cannot be covered and contained within a clean, dry bandage.^83^

**34.** *(unanimous consensus)* Inform caregivers that disclosure of a child’s *C. auris* colonization status to personnel in non-healthcare congregate settings should occur only with their consent.^27,49^

**35.** *(consensus)* Limit disclosure to personnel who may provide medical care, including device care, or those who supervise or perform cleaning and disinfection of uncontrolled excretions (eg, vomit) or diarrhea from children with *C. auris* colonization.^49^

Remarks for **34** and **35**:

- Indiscriminate disclosure of colonization status can lead to stigmatization, exclusion, and improper patient isolation and implementation of Contact Precautions.^27,49,50^
- Public health partners may request disclosure for certain scenarios, including for communities with high prevalence or known transmission of *C. auris*.
- Consensus achieved with 90% agreement (18 of 20 panelists agreed, 2 disagreed and 0 abstained).

**36.** *(unanimous consensus)* Educate HCP to wear gloves and gowns if there is a risk of soiling with body fluids while providing medical care to children with *C. auris* colonization.^86^

**37.** *(unanimous consensus)* Educate HCP to use an EPA-registered product effective against *C. auris* for cleaning and disinfection of high-touch surfaces after providing medical care, including device care, or of areas soiled by uncontrolled excretions.^2,39^

Remarks for **36** and **37**:

- During times of transmission of *C. auris* in non-healthcare congregate settings, partners in public health may instruct staff to implement IPC strategies, including hand hygiene, PPE, and environmental controls.
- HCP performing device care for children with *C. auris* colonization may perform device care for other children. The risk of *C. auris* transmission is reduced by hand hygiene, wearing PPE, and environmental disinfection.^2^

**38.** *(consensus)* Do not screen classmates or peers for *C. auris* colonization *(expert opinion)*.

Remarks:

- During times of high prevalence or suspected transmission of *C. auris*, partners in public health may request surveillance, risk assessment, or implementation of IPC strategies including hand hygiene, PPE, and environmental controls.
- Consensus achieved with 95% agreement (19 of 20 panelists agreed, 0 disagreed and 1 abstained).

See Supplementary Material Table 3 for a list of future research needs for prevention of *C. auris* in pediatric settings.

## Supporting information

10.1017/ash.2026.10419.sm001 Murray et al. supplementary material 1 Murray et al. supplementary material

10.1017/ash.2026.10419.sm002 Murray et al. supplementary material 2 Murray et al. supplementary material

10.1017/ash.2026.10419.sm003 Murray et al. supplementary material 3 Murray et al. supplementary material

## Supplementary material

The supplementary material for this article can be found at https://doi.org/10.1017/ash.2026.10419.
