# Supplementary material for: Infection prevention and control of Candida auris in pediatric settings
Source: Antimicrob Steward Healthc Epidemiol. 2026 Jun 23;6(1):e183. doi: 10.1017/ash.2026.10419 (PMC13312240; doi:10.1017/ash.2026.10419)
Supplement: Murray et al. supplementary material 1 — Murray et al. supplementary material [file S2732494X26104197sup001.pdf]

Supplementary Materials

Table 1. General recommendations and additional pediatric recommendations for infection prevention and control (IPC) of *C. auris*

Acute healthcare settings
**Public health and facility response**
Given the relative rarity of *C. auris* in the pediatric population, collaboration and communication with local IPC (see **SM Table 2**) and public health partners are essential[1-3].<br><br>**General recommendations**<br>• Report possible or confirmed cases of *C. auris* infection immediately to the clinical team, local IPC, and public health partners. Reporting thresholds may vary by jurisdiction resulting in different recommendations in certain circumstances[1,2,4]; however, healthcare facilities should have a low threshold for reporting cases in pediatric patients to public health partners.<br>• Collaborate with local IPC and public health partners to receive guidance and determine the response for newly identified cases of *C. auris,* suspected or confirmed transmission of *C. auris* in a healthcare facility, or high rates of local community transmission[1-3].
**No additional pediatric recommendations.**
**Identifying patient risk factors and risk mitigation**
**General recommendations**<br>• Identify the patient's risk factors for *C. auris* colonization or infection which may include[5-8]:<br>○ Known epidemiologic link to *C. auris* and/or clinical risk factors:<br>▪ Exposure in a healthcare facility, e.g., sharing the same room, spending time in a shared care area with a patient with *C. auris* even if the person with *C. auris* has been discharged or is no longer in the care area, or using shared mobile equipment as a patient with *C. auris* colonization or infection.<br>▪ Care in a healthcare facility with current, suspected, or confirmed *C. auris* transmission or in a community with high *C. auris* transmission rates.<br>▪ Transfer process should include information about current, suspected, or confirmed *C. auris* transmission.<br>○ Clinical factors:<br>▪ Presence of an indwelling medical device (e.g., CVC, tracheostomy tube, gastrostomy tube, urinary catheter), mechanical ventilation, or the use of parenteral nutrition.<br>▪ Treatment with broad spectrum antimicrobials such as third generation cephalosporins or carbapenem agents or prolonged antifungal prophylaxis.<br>• Risk mitigation:<br>○ Assess the need for all modifiable risk factors daily, including use of indwelling medical device(s) and antimicrobials[8-12].

- Adhere to care bundles for medical device care[9-11].

No additional pediatric recommendations. *See **Background, Risk Factors** in the manuscript for discussion of risk factors unique to pediatric populations, including:*

- Epidemiologic risk factors:
  - Frequent or prolonged inpatient hospitalizations
  - Sharing a room, care area, or mobile equipment with a patient colonized or infected with *C. auris*
  - Receipt of care in a healthcare with suspected, confirmed, or ongoing *C. auris* transmission
  - Residence in a community with high *C. auris* transmission rates
- Potential additional risk factors for pediatric patients:
  - Having a birth parent or caregiver colonized or infected with *C. auris*
  - Being breastfed by a caregiver colonized with *C. auris*
- Clinical risk factors:
  - Presence of indwelling medical devices
  - Mechanical ventilation
  - Receipt of parenteral nutrition
  - Exposure to broad spectrum antimicrobials
  - Prolonged antifungal prophylaxis
- Clinical risk factors for pediatric patients:
  - Extreme prematurity
  - Congenital heart disease
  - Malnutrition
- Sharing space, equipment, or diagnostic areas with patients, including adult patients, colonized or infected with *C. auris*.

Education, training, monitoring, and adherence

At the time of developing these recommendations, *C. auris* infection or colonization remains relatively rare in pediatrics. HCP, caregivers, and other close contacts (see **SM Table 2**), e.g., household members, may be unaware of this pathogen and unfamiliar with strategies to prevent transmission in pediatric healthcare and non-healthcare settings[13].

General recommendations

- Provide HCP and support staff in facilities with suspected, confirmed, or ongoing *C. auris* transmission with regular education and training on prevention of *C. auris*[14]. Include the following[13,15,16]:
  - Risks of environmental transmission of *C. auris*
  - Proper cleaning and disinfection protocols
  - IPC protocols
  - When to suspect *C. auris* based on epidemiologic risk factors and clinical context and when to notify local IPC or infectious diseases specialists.
- Monitor adherence to IPC protocols and provide feedback to staff and leadership[15,17].

Additional pediatric recommendations

1. *(unanimous consensus)* Educate the caregivers, family members, and visitors of a child in an acute care setting who is infected or colonized with *C. auris* about strategies to reduce the risk of acquisition or transmission of *C. auris* during hospitalization[15,17]. Include the following education:

- Components of Standard Precautions (see **SM Table 2**)
- When and how to perform hand hygiene
- When and how to safely don and doff personal protective equipment (PPE), especially gloves and gowns.

2. *(unanimous consensus)* Educate caregivers and visitors who are known to be colonized or infected with *C. auris* about strategies to reduce transmission to the hospitalized pediatric patient and reduce transmission to close contacts after discharge (see **Recommendation 1** for topics of education)[15,17,18].

3. *(consensus)* Provide caregivers with discharge information on *C. auris* in plain language that includes disclosure information and strategies to prevent transmission of *C. auris* in healthcare and non-healthcare congregate settings[15,19-21].

Surveillance and screening

Currently, approaches to surveillance and screening for *C. auris* vary across settings and epidemiologic context and are not standardized [22]. Screening may be used to determine if exposed patients (see **SM Table 2**) have become colonized.

General recommendations

- Place exposed patients who have a positive screening test for *C. auris* on Contact Precautions.
- When a facility suspects or identifies ongoing *C. auris* transmission, perform point prevalence surveys every 7 to 14 days on the entire unit, not just sub-sections of the unit[3,23,24].
  - Reduce the frequency of the surveys as transmission decreases[3].
- Perform contact tracing of additional patients (not on the unit) at high risk for *C. auris* and include in point prevalence surveys (see **Risk Factors**)[2,3,5,6,8,25].
- Do not re-screen patients with known *C. auris* colonization or infection to assess their status or to remove Contact Precautions[8].
- Do not screen healthcare personnel (HCP) for *C. auris*[8].

Additional pediatric recommendations

4. *(unanimous consensus)* Do not screen patients admitted to the NICU or other acute units unless one of following known exposures to *C. auris* exists (*expert consensus*):

- A birth parent, caregiver, or other close contact is colonized or infected with *C. auris*.
- The patient is breastfed by a caregiver who is colonized or infected with *C. auris*.
- The patient is sharing space or equipment with patients, including adult patients, who have *C. auris* colonization or infection.
- The patient was transferred from a facility with suspected, confirmed, or ongoing *C. auris* transmission.
- Patients who are sharing space or equipment with adult patients who have *C. auris* colonization or infection.

5. *(consensus)* For an infant in the NICU whose birth parent is colonized or infected with *C. auris*, screen the infant to inform IPC decisions and empiric antimicrobial therapy, using the following recommended screening intervals[26-28]:

- At birth or after bathing
- On the seventh day of life if the neonate (see **SM Table 2**) remains hospitalized
- Every 2 weeks thereafter if the infant remains hospitalized

- As needed to inform decision-making
- At hospital discharge
- At readmission.

6. *(consensus)* Do not screen caregivers and other close contacts of pediatric patients colonized or infected with *C. auris* unless the caregiver or other close contact requires frequent inpatient healthcare interactions[2,3,8,29].

Swab collection

General recommendations

- Obtain a composite swab of the patient's bilateral axilla and groin[30].

Additional pediatric recommendations

7. *(majority opinion)* Obtain a bilateral composite swab of the patient's nares, axilla, and groin[8,28,31,32].

Cultures and PCR

General recommendations

- Depending on local resources, real-time polymerase chain reaction (PCR) is the preferred method for detecting colonization but may detect non-viable organisms[33,34].
- If performing culture, use a *Candida* chromogenic medium and incubate at the temperature stated in the manufacturers' instructions for use (MIFU) to recover isolates for species confirmation[33].
- Current Matrix-Assisted Laser Desorption/Ionization Time-of-Flight Mass Spectrometry (MALDI-TOF MS) systems can reliably identify isolates of *C. auris*. Variable performance has been reported for biochemical test systems[34].
- Depending on local resources, facilities may process swab specimens for *C. auris* culture with or without broth enrichment or perform PCR directly from swab specimen[33].
- Local IPC in facilities without the capacity to perform *C. auris* screening should coordinate with county or state public health departments to perform screening testing in a reference laboratory or a public health laboratory. The CDC Antimicrobial Resistance Laboratory Network offers free PCR-based colonization testing for healthcare facilities and health departments[8].

No additional pediatric recommendations.

Contact Precautions and hand hygiene

General recommendations

- Implement Contact and Standard Precautions for patients infected or colonized with *C. auris*[15].
- Ensure HCP, caregivers, and visitors perform hand hygiene before and after entering the room of a patient infected or colonized with *C. auris* and before and after direct contact with a patient or their environment or items[15].
- Perform hand hygiene using alcohol-based hand sanitizer or soap and water when hands are visibly soiled[3,15,35,36].
- Gloves are not a substitute for hand hygiene[15,35,36]. Perform hand hygiene before putting on gloves and after removing gloves.
- Maintain Contact Precautions for the duration of hospitalization and during future acute care admissions[8,15].

Additional pediatric recommendations

<u>Infants and children</u> colonized or infected with *C. auris*:
8. *(consensus)* Implement Contact and Standard Precautions for *C. auris* per local IPC and public health recommendations *(expert opinion)*.
9. *(consensus)* Consult local IPC and public health partners to determine the duration of Contact Precautions for a pediatric patient's current and future admissions to acute care (*expert opinion*).

<u>Caregivers</u> colonized or infected with *C. auris*:
10. *(consensus)* Place hospitalized newborns, infants (including those in the NICU), and children on Contact Precautions if their caregiver is known to be colonized or infected with *C. auris* (*expert opinion*).
11. *(consensus)* If known, document the caregiver's colonization or infection with *C. auris* in the patient's medical record for implementation of Contact Precautions for *C. auris* for the patient in the case of future healthcare admissions, including non-acute healthcare facilities (*expert opinion*).
12. *(consensus)* For current and future healthcare admissions, including long-term care, consult local IPC and public health partners to determine the appropriate duration for Contact Precautions for the child of a caregiver who is colonized or infected with *C. auris* (*expert opinion*).

Room placement and cohorting

General recommendations

- Place a patient who has *C. auris* colonization or infection in a single-patient room with a dedicated bathroom, whenever possible[15].
- If single rooms are unavailable, cohort patients together who have *C. auris* colonization or infection[15,23].

Additional pediatric recommendations
<u>Infants and children</u> colonized or infected with *C. auris*:
13. *(consensus)* Allow caregivers to room-in with pediatric patients colonized or infected with *C. auris*[37,38].
14. *(consensus)* For NICUs with open bays or pods, follow local IPC and public health partner guidance for placement of isolettes or cribs of infants with *C. auris* colonization or infection (*expert opinion*).

<u>Caregivers</u> colonized or infected with *C. auris*:
15. *(unanimous consensus)* Allow caregivers who are colonized with *C. auris* to room-in with their child[37,38].
16. *(consensus)* In accordance with local IPC guidance, before a caregiver with active *C. auris* infection rooms-in with a pediatric patient, confirm that they have received appropriate antifungal treatment and are showing signs of improvement[32].

Breastfeeding and skin-to-skin practices

No general recommendations.

Pediatric recommendations
<u>Infants and children</u> colonized or infected with *C. auris*:
17. *(unanimous consensus)* Encourage and facilitate breastfeeding and skin-to-skin practices[39-43].
18. *(unanimous consensus)* Use shared decision-making (see **SM Table 2**) to guide breastfeeding and skin-to-skin practices for caregivers with underlying medical conditions or frequent inpatient healthcare exposures (*expert opinion*).

<u>Caregivers</u> who are colonized or infected with *C. auris*:

19. *(consensus)* Use shared medical decision-making to guide breastfeeding and skin-to-skin practices for caregivers who are colonized or infected with *C. auris*, including decisions about cleansing the caregiver's breasts or skin with soap and water before breastfeeding or skin-to-skin practices (*expert opinion*).
20. *(consensus)* Instruct a caregiver with *C. auris* infection involving the milk ducts or an active cutaneous lesion involving the breast to avoid breastfeeding or to discard EBM (*expert opinion*).
21. *(consensus)* Instruct a caregiver with *C. auris* infection to prevent the infant from contacting areas with active infection (*expert opinion*).

Visitation and common spaces for acute care settings

No general recommendations.

Pediatric recommendations

22. *(consensus)* Consult local IPC to develop policies for pediatric patients with *C. auris* colonization or infection, including patients in the NICU or the newborn nursery, that address the following (*expert opinion*):

- Visitation by siblings
- Visitation by caregivers or visitors who are colonized or infected with *C. auris*
- PPE use by caregivers who are rooming in
- PPE use by visitors who are not rooming in
- Temporary transport to shared care areas (e.g., procedural areas)
- Appropriate use of common spaces (e.g., kitchens, washing machines, lounges, child life areas) by caregivers, visitors, and siblings, including any necessary restrictions or mitigation strategies.

Decolonization

General recommendations

- Currently, no agent has been identified to be effective and efforts to decolonize do not prevent transmission of *C. auris*[2,15].
- Emphasize personal hygiene, especially of known colonized skin areas (such as the axillae, inguinal folds, palms) which can decrease the bioburden of *C. auris* and therefore reduce the likelihood of transmission[44].
- The effects of chlorhexidine on reducing skin burden or infection with *C. auris* have not been systematically studied[13,15].

No additional pediatric recommendations.

Supplies, medical and non-medical equipment, and environmental cleaning and disinfection

General recommendations

- For patients with *C. auris* colonization or infection[3,13,15]
    - Use single-patient or disposable equipment whenever possible.
    - Use single-patient supplies, such as sterile ultrasound gel packets.
    - Use impermeable, sealed bags to handle linens and waste
    - Use standard healthcare laundering.
- At least daily, clean and disinfect the rooms of patients with *C. auris* colonization or infection with an EPA-registered product effective against *C. auris*[15,45,46]

- Terminally clean shared spaces (e.g., occupational and physical therapy areas) that were used by patients with *C. auris* colonization or infection[15].
- Clean and disinfect medical equipment after each use by patients with *C. auris* colonization or infection, e.g., glucometers, ventilators, ultrasound machines, blood pressure cuffs[15,47].
- Label disinfected medical equipment and separate it from dirty equipment[15].
- For disinfection of rooms and mobile and reusable equipment, use EPA-registered products (see **SM Table 2**) effective against *C. auris* (List P)[2,15,48].
- Follow the MIFU for disinfectants' contact times[48].
- Data on "no touch" devices such as germicidal UV irradiation and vaporized hydrogen peroxide are limited and parameters for effective disinfection are not well understood. Facilities may use these supplemental methods only after completing recommended standard cleaning and disinfection[15].

Additional pediatric recommendations

23. *(consensus)* Follow disinfection requirements for specialized pediatric equipment, including items with complex MIFUs, such as incubator and warmer beds, isolettes, milk warmers, X-ray plates and machines, ultrasound probes and machines, ophthalmology equipment, and audiology equipment[13,49].

24. *(consensus)* Select non-medical equipment (e.g., toys, tablets, video games consoles, and controllers) that are compatible with an EPA-registered product effective against *C. auris* and establish cleaning and disinfecting protocols with local IPC and child life specialists (*expert opinion*).

25. *(consensus)* If possible, dedicate toys for exclusive use by an infant or child on Contact Precautions for *C. auris*[50].

26. *(consensus)* For caregivers colonized or infected with *C. auris*, and for infants colonized or infected with *C. auris*, when possible, dedicate breast pumps and accessories for exclusive use by the breastfeeding caregiver to use in the infant's room. Clean and disinfect the pump and accessories after each use with an EPA-registered product effective against *C. auris*[48,51].

27. *(consensus)* For pediatric patients whose caregiver is *C. auris* colonized or infected, clean and disinfect the patient's room and medical and non-medical equipment (see **Recommendation 26**) at least daily (*expert opinion*).

Communication and documentation

General recommendations

- Place alerts in the medical record that indicate patients with *C. auris* colonization or infection[15].
- Place visible signage for Contact Precautions outside the patient's room[15].
- Limit transport of patients with *C. auris* colonization or infection to medically necessary procedures[15].
- To ensure continuity of Contact Precautions, notify receiving departments or facilities of a patient's *C. auris* status[15].

No additional pediatric recommendations.

Non-acute healthcare settings

General recommendations

Comprehensive recommendations for nursing homes caring for adult patient populations have been published[52].

Pediatric recommendations

28. *(consensus)* Do not exclude infants and children with *C. auris* colonization or infection from pediatric non-acute healthcare settings[53].

29. *(consensus)* Consult local IPC and public health partners to determine the duration of Contact Precautions for a pediatric resident's current and future admissions to non-acute care facilities (*expert opinion*).
30. *(consensus)* Use Standard Precautions with an emphasis on hand hygiene when caring for residents with *C. auris* colonization during low- or moderate-risk activities involving controlled or minimal contact with secretions or excretions (e.g., school interactions, diaper changes without diarrhea, assistance with eating, or localized wound care) (*expert opinion*).
31. *(consensus)* Use Enhanced Barrier Precautions (gloves and gowns; see **SM Table 2**) when caring for residents during higher risk activities that involve extended contact with secretions or excretions (e.g., manipulating medical devices, bathing, or changing the diaper of a resident with diarrhea)[19,54].
32. *(consensus)* Consult local IPC and public health partners, when relevant, to develop visitation policies for: (*expert opinion*)

- Pediatric residents who are colonized or infected with *C. auris*
- Sibling visitation for pediatric residents who are colonized or infected with *C. auris*
- Caregivers or visitors who are colonized or infected with *C. auris*.

Non-healthcare congregate settings

No general recommendations.

Pediatric recommendations

33. *(unanimous consensus)* Do not exclude children colonized with *C. auris* from non-healthcare congregate settings[53].
34. *(unanimous consensus)* Inform caregivers that disclosure of a child's *C. auris* colonization status to personnel in non-healthcare congregate settings should occur only with their consent[19,20].
35. *(consensus)* Limit disclosure to personnel who may provide medical care, including device care, or those who supervise or perform cleaning and disinfection of uncontrolled excretions (e.g., vomit or diarrhea) from children with *C. auris* colonization[20].
36. *(unanimous consensus)* Educate HCP to wear gloves and gowns if there is a risk of soiling with body fluids while providing medical care to children with *C. auris* colonization[55].
37. *(unanimous consensus)* Educate HCP to use an EPA-registered product effective against *C. auris* for cleaning and disinfection of high-touch surfaces after providing medical care, including device care, or of areas soiled by uncontrolled excretions[3,15].
38. *(consensus)* Do not screen classmates or peers for *C. auris* colonization *(expert opinion)*.

References

1. Greco C, Smith H, Gilbert B, et al. Candida auris inpatient screening in collaboration with the public health department. *Am J Infect Control*. Aug 2024;52(8):981-983. doi:10.1016/j.ajic.2024.05.010
2. California Department of Public Health (CDPH) Healthcare-Associated Infections Program. *Candida auris* Quicksheet. https://www.cdph.ca.gov/Programs/CHCQ/HAI/CDPH%20Document%20Library/CaurisQuicksheet.pdf
3. Centers for Disease Control and Prevention. MDRO containment strategy. https://www.cdc.gov/healthcare-associated-infections/php/preventing-mdros/mdro-containment-strategy.html
4. Centers for Disease Control and Prevention. Reportable Fungal Diseases by State. Feb. 10, 2026. Accessed Feb. 10, 2026. https://www.cdc.gov/fungal/php/case-reporting/index.html

5. Alvarado-Socarras JL, Vargas-Soler JA, Franco-Paredes C, Villegas-Lamus KC, Rojas-Torres JP, Rodriguez-Morales AJ. A Cluster of Neonatal Infections Caused by Candida auris at a Large Referral Center in Colombia. *J Pediatric Infect Dis Soc*. May 28 2021;10(5):549-555. doi:10.1093/jpids/piaa152
6. Danielsen AS, Odeskaug LE, Raastad R, et al. Key Factors to Consider for Candida auris Screening in Healthcare Settings: A Systematic Review. *Mycoses*. Mar 2025;68(3):e70043. doi:10.1111/myc.70043
7. Centers for Disease Control and Prevention. Preventing the Spread of C. auris. CDC. Feb. 3, 2026, Updated Dec. 15, 2025. Accessed Feb. 1, 2026. https://www.cdc.gov/candida-auris/prevention/index.html
8. Centers for Disease Control and Prevention. Screening recommendations for healthcare facilities. https://www.cdc.gov/candida-auris/hcp/screening-hcp/index.html
9. Buetti N, Marschall J, Drees M, et al. Strategies to prevent central line-associated bloodstream infections in acute-care hospitals: 2022 Update. *Infect Control Hosp Epidemiol*. May 2022;43(5):553-569. doi:10.1017/ice.2022.87
10. Klompas M, Branson R, Cawcutt K, et al. Strategies to prevent ventilator-associated pneumonia, ventilator-associated events, and nonventilator hospital-acquired pneumonia in acute-care hospitals: 2022 Update. *Infect Control Hosp Epidemiol*. Jun 2022;43(6):687-713. doi:10.1017/ice.2022.88
11. Patel PK, Advani SD, Kofman AD, et al. Strategies to prevent catheter-associated urinary tract infections in acute-care hospitals: 2022 Update. *Infect Control Hosp Epidemiol*. Aug 2023;44(8):1209-1231. doi:10.1017/ice.2023.137
12. Centers for Disease Control and Prevention. Core Elements of Hospital Antibiotic Stewardship Programs. Updated Sept. 15, 2025. Accessed Feb. 1, 2026. https://www.cdc.gov/antibiotic-use/hcp/core-elements/hospital.html
13. UK Health Security Agency. *Candidozyma auris* guidance. Infection prevention and control (IPC). https://www.gov.uk/government/publications/candida-auris-laboratory-investigation-management-and-infection-prevention-and-control/infection-prevention-and-control-ipc
14. Popovich KJ, Aureden K, Ham DC, et al. SHEA/IDSA/APIC Practice Recommendation: Strategies to prevent methicillin-resistant Staphylococcus aureus transmission and infection in acute-care hospitals: 2022 Update. *Infect Control Hosp Epidemiol*. Jul 2023;44(7):1039-1067. doi:10.1017/ice.2023.102
15. Centers for Disease Control and Prevention. Infection Control Guidance: *Candida auris*: Setting-Based Precautions. https://www.cdc.gov/candida-auris/hcp/infection-control/
16. Centers for Disease Control and Prevention. Candida auris in Health Care: Recognize the Risk and Stop the Spread. Feb. 3, 2026. Updated Dec. 29, 2025. Accessed Feb. 3, 2026. https://www.cdc.gov/project-firstline/hcp/training/c-auris.html
17. Government of Canada, Public Health Agency of Canada. *Candida auris* Infection Prevention and Control in Canadian Healthcare Settings. https://www.canada.ca/en/services/health/publications/diseases-conditions/candida-auris-infection-prevention-control-canadian-healthcare-settings.html
18. UK Health Security Agency. Candidozyma auris: information for patients and visitors. Feb. 10, 2026. Accessed Feb. 10, 2026. https://www.gov.uk/guidance/candidozyma-auris-information-for-patients-and-visitors
19. Guzman-Cottrill JA, Blatt DB, Bryant KA, et al. SHEA practice update: infection prevention and control (IPC) in residential facilities for pediatric patients and their families. *Infect Control Hosp Epidemiol*. Nov 14 2024;46(1):1-24. doi:10.1017/ice.2024.124

20. American Academy of Pediatrics. Managing Infectious Diseases in Child Care and Schools: A Quick Reference Guide. In: Shope TR, Hashikawa AN, eds. American Academy of Pediatrics; 2023:1-14:chap Overview of Managing Infectious Diseases in Child Care and Schools.
21. Rathore MH, Jackson MA. Infection Prevention and Control in Pediatric Ambulatory Settings. *Pediatrics*. Nov 2017;140(5)doi:10.1542/peds.2017-2857
22. Hennessee IP, Forsberg K, Beekmann SE, Polgreen PM, Gold JAW, Lyman M. Candida auris screening practices at healthcare facilities in the United States: An Emerging Infections Network survey. *Infect Control Hosp Epidemiol*. Jun 2024;45(6):766-769. doi:10.1017/ice.2024.5
23. Shuping L, Maphanga TG, Naicker SD, et al. High Prevalence of Candida auris Colonization during Protracted Neonatal Unit Outbreak, South Africa. *Emerg Infect Dis*. Sep 2023;29(9):1913-1916. doi:10.3201/eid2909.230393
24. Aldejohann AM, Wiese-Posselt M, Gastmeier P, Kurzai O. Expert recommendations for prevention and management of Candida auris transmission. *Mycoses*. Jun 2022;65(6):590-598. doi:10.1111/myc.13445
25. McPherson TD, Walblay KA, Roop E, et al. Notes from the Field: Candida auris and Carbapenemase-Producing Organism Prevalence in a Pediatric Hospital Providing Long-Term Transitional Care - Chicago, Illinois, 2019. *MMWR Morb Mortal Wkly Rep*. Aug 28 2020;69(34):1180-1181. doi:10.15585/mmwr.mm6934a5
26. Magnasco L, Mikulska M, Sepulcri C, et al. Frequency of Detection of Candida auris Colonization Outside a Highly Endemic Setting: What Is the Optimal Strategy for Screening of Carriage? *Journal of fungi (Basel, Switzerland)*. Dec 29 2023;10(1)doi:10.3390/jof10010026
27. Mesini A, Saffioti C, Mariani M, et al. First Case of Candida auris Colonization in a Preterm, Extremely Low-Birth-Weight Newborn after Vaginal Delivery. *Journal of fungi (Basel, Switzerland)*. Aug 10 2021;7(8)doi:10.3390/jof7080649
28. Biswal M, Rudramurthy SM, Jain N, et al. Controlling a possible outbreak of Candida auris infection: lessons learnt from multiple interventions. *Journal of Hospital Infection*. 2017/12/01/ 2017;97(4):363-370. doi:https://doi.org/10.1016/j.jhin.2017.09.009
29. UK Health Security Agency. *Candidozyma auris:* guidance. Screening of patients and contacts. https://www.gov.uk/government/publications/candida-auris-laboratory-investigation-management-and-infection-prevention-and-control/screening-of-patients-and-contacts#contents
30. Centers for Disease Control and Prevention. *C. auris* screening: Patient swab collection. https://www.cdc.gov/candida-auris/hcp/screening-hcp/c-auris-screening-patient-swab-collection-1.html
31. Sansom SE, Gussin GM, Schoeny M, et al. Rapid Environmental Contamination With Candida auris and Multidrug-Resistant Bacterial Pathogens Near Colonized Patients. *Clin Infect Dis*. May 15 2024;78(5):1276-1284. doi:10.1093/cid/ciad752
32. Banach DB, Bearman GM, Morgan DJ, Munoz-Price LS. Infection control precautions for visitors to healthcare facilities. *Expert review of anti-infective therapy*. 2015;13(9):1047-50. doi:10.1586/14787210.2015.1068119
33. Korsten K, Gerrits van den Ende B, Pique RD, Hagen F, van Dijk K. Keep the Hospital Clean: Diagnostic Performance of Ten Different Molecular and Culture-Based Methods to Detect Candidozyma (Candida) auris. *Mycopathologia*. Apr 15 2025;190(3):37. doi:10.1007/s11046-025-00944-8
34. Orner EP, Thwe PM. Candida auris Diagnostics: Identification and Screening. *Clin Lab Med*. Mar 2025;45(1):101-110. doi:10.1016/j.cll.2024.10.005
35. World Health Organization Patient Safety. *WHO Guidelines on Hand Hygiene in Health Care*. World Health Organization. https://iris.who.int/server/api/core/bitstreams/b7cdc469-d662-4958-adfd-949a750e5ad9/content

36. Boyce JM, Pittet D, Healthcare Infection Control Practices Advisory C, Force HSAIHHT. Guideline for Hand Hygiene in Health-Care Settings. Recommendations of the Healthcare Infection Control Practices Advisory Committee and the HICPAC/SHEA/APIC/IDSA Hand Hygiene Task Force. Society for Healthcare Epidemiology of America/Association for Professionals in Infection Control/Infectious Diseases Society of America. *MMWR Recomm Rep*. Oct 25 2002;51(RR-16):1-45, quiz CE1-4.
37. Munoz-Price LS, Banach DB, Bearman G, et al. Isolation precautions for visitors. *Infection Control & Hospital Epidemiology*. 2015;36(7):747-758. doi:10.1017/ice.2015.67
38. Darby J, Falco C. Infection control and the need for family-/child-centered care. In: McNeil JC, Campbell JR, Crews JD, eds. Springer Nature; 2018:57-79:chap Healthcare-Associated Infections in Children.
39. Parker MG, Stellwagen L, Miller ER, et al. Promoting Human Milk and Breastfeeding for the Very Low Birth Weight Infant: Clinical Report. *Pediatrics*. Jan 12 2026;doi:10.1542/peds.2025-073625
40. Akinboyo IC, Zangwill KM, Berg WM, Cantey JB, Huizinga B, Milstone AM. SHEA neonatal intensive care unit (NICU) white paper series: Practical approaches to Staphylococcus aureus disease prevention. *Infect Control Hosp Epidemiol*. Nov 2020;41(11):1251-1257. doi:10.1017/ice.2020.51
41. Patnode CD, Henrikson NB, Webber EM, Blasi PR, Senger CA, Guirguis-Blake JM. Breastfeeding and Health Outcomes for Infants and Children: A Systematic Review. *Pediatrics*. Jul 1 2025;156(1)doi:10.1542/peds.2025-071516
42. Hendricks-Munoz KD, Xu J, Parikh HI, et al. Skin-to-Skin Care and the Development of the Preterm Infant Oral Microbiome. *Am J Perinatol*. Nov 2015;32(13):1205-16. doi:10.1055/s-0035-1552941
43. Davis EC, Castagna VP, Sela DA, et al. Gut microbiome and breast-feeding: Implications for early immune development. *The Journal of allergy and clinical immunology*. Sep 2022;150(3):523-534. doi:10.1016/j.jaci.2022.07.014
44. Ashkenazi-Hoffnung L, Rosenberg Danziger C. Navigating the New Reality: A Review of the Epidemiological, Clinical, and Microbiological Characteristics of Candida auris, with a Focus on Children. *Journal of fungi (Basel, Switzerland)*. Jan 28 2023;9(2)doi:10.3390/jof9020176
45. Adams E, Quinn M, Tsay S, et al. Candida auris in Healthcare Facilities, New York, USA, 2013-2017. *Emerg Infect Dis*. Oct 2018;24(10):1816-1824. doi:10.3201/eid2410.180649
46. Tsay S, Welsh RM, Adams EH, et al. Notes from the Field: Ongoing Transmission of Candida auris in Health Care Facilities - United States, June 2016-May 2017. *MMWR Morb Mortal Wkly Rep*. May 19 2017;66(19):514-515. doi:10.15585/mmwr.mm6619a7
47. Centers for Disease Control and Prevention. Infection Control Actions for Prevention and Response. Response to *C. auris* confirmation. https://www.cdc.gov/candida-auris/hcp/infection-control/prevention-response.html#cdc_generic_section_2-response-to-c-auris-confirmation
48. US Environmental Protection Agency. EPA's Registered Antimicrobial Products Effective Against *Candida auris*. https://www.epa.gov/pesticide-registration/epas-registered-antimicrobial-products-effective-against-candida-auris-list
49. AAP-COFN, ACOG. *Guidelines for Perinatal Care*. 8 ed. AAP-COFN
ACOG; 2017:691.
50. Koutlakis-Barron I, Hayden TA. Essentials of infection prevention in the pediatric population. *Int J Pediatr Adolesc Med*. Dec 2016;3(4):143-152. doi:10.1016/j.ijpam.2016.10.002

51. Centers for Disease Control and Prevention. Environmental Cleaning Procedures. Best Practices for Environmental Cleaning in Healthcare Facilities. Feb. 10, 2026. Updated Mar. 19, 2024. https://www.cdc.gov/healthcare-associated-infections/hcp/cleaning-global/procedures.html?CDC_AAref_Val=https://www.cdc.gov/hai/prevent/resource-limited/cleaning-procedures.html
52. Mody L, Advani SD, Ashraf MS, et al. Multisociety guidance for infection prevention and control in nursing homes. *Infect Control Hosp Epidemiol*. Oct 28 2025;46(11):1-28. doi:10.1017/ice.2025.10252
53. Centers for Disease Control and Prevention. Schools and daycares: MRSA prevention and response. https://www.cdc.gov/mrsa/prevention/schools-and-daycares.html
54. Centers for Disease Control and Prevention. Consideration for Use of Enhanced Barrier Precautions in Skilled Nursing Facilities. https://www.cdc.gov/infection-control/media/pdfs/enhancedbarrierprecautions-508.pdf?
55. Centers for Disease Control and Prevention. Implementation of personal protective equipment (PPE) use in nursing homes to prevent spread of multidrug-resistant organisms (MDROs). https://www.cdc.gov/long-term-care-facilities/hcp/prevent-mdro/ppe.html
