# Supplementary material for: Infection prevention and control of Candida auris in pediatric settings
Source: Antimicrob Steward Healthc Epidemiol. 2026 Jun 23;6(1):e183. doi: 10.1017/ash.2026.10419 (PMC13312240; doi:10.1017/ash.2026.10419)
Supplement: Murray et al. supplementary material 2 — Murray et al. supplementary material [file S2732494X26104197sup002.pdf]

Supplementary Materials

Table 2. Terminology

Term	Definition
Additional pediatric recommendations	In this document, *additional pediatric recommendations* apply to a wide range of settings including well baby nurseries, neonatal intensive care units, hospitalized children, children residing in long term care facilities, and children attending non-healthcare congregate settings.
Candida auris (*C. auris*)	*C. auris* is a multidrug-resistant fungal pathogen causing healthcare-associated infections and outbreaks worldwide with substantial morbidity and mortality.<br><br>*Candida auris* (*C. auris*) is used in this document to facilitate clear clinical recognition and communication, while acknowledging that *Candidozyma auris* has been proposed as a taxonomic revision to *Candida auris* and is used in some literature and guidance.
Caregiver	Per the SHEA Handbook, “a visitor who is a primary source of support for the patient’s activities of daily living, labor and delivery, or a procedure and recovery.” In this document, “caregiver” may refer to a birth parent, adoptive parent, guardian, or other primary caregiver, e.g., a grandparent.[1]
Cleaning	Procedures to remove soiling of equipment or the environment prior to disinfection, if required.
Close contacts	Individuals other than the primary caregiver who have direct interactions with the pediatric patient who is colonized or infected with *C. auris*, such as household members, members of the child’s extended family, or intimate partners. Examples of direct interactions include hugging, kissing, or touching.
Colonized	Individual who is asymptomatic with a positive culture or a molecular test for *C. auris*[2].
Contact Precautions	IPC measures implemented in healthcare settings for patients who are known or suspected to be infected or colonized with pathogens transmitted by direct or indirect contact to prevent transmission from the patient or from their contaminated environment.<br><br>Elements of Contact Precautions include: placement in single-patient room, whenever available; use of personal protective equipment (PPE) (i.e., gowns and gloves); dedicated or disposable medical equipment; limiting transport and movement of patients outside of their room except for medically necessary purposes; and prioritized cleaning and disinfection of patients’ rooms and bathrooms at least daily with a focus on frequently touched surfaces and equipment.
Decolonization	Treatment to reduce or eliminate the burden of specific, often antimicrobial-resistant bacteria (like MRSA or *C. auris*) from a patient’s body, typically from the nose, skin, or gut using antiseptic agents (e.g., chlorhexidine) or antibiotics.
Disinfection	Procedures to eliminate pathogenic microorganisms on inanimate objects, using high, intermediate, or low-level processes based on equipment type and use[3].
Enhanced Barrier Precautions	IPC measures implemented in non-acute healthcare settings, e.g., long-term care facilities, to reduce transmission of multidrug-resistant organisms (MDROs) from residents known to be colonized or infected with MDROs or residents at increased risk of acquiring MDROs, e.g., those with wounds or indwelling devices.<br>• Elements of Enhanced Barrier Precautions include the use of gowns and gloves during high-contact activities with residents.<br>• Examples of high-contact activities with residents by HCP and staff include dressing, bathing/showering, transferring, providing hygiene, changing linens, changing briefs, assisting with toileting, device care or use, and wound care.

Enhanced environmental disinfection	Increased frequency of cleaning and disinfection when there is concern for environmental contamination, e.g., the hospital room of a patient with *C. auris* who is colonized or infected with *C. auris*.
Exposed	Individual who has 1) an epidemiological link to another individual who is colonized or infected with *C. auris*, e.g., from a caregiver or household member colonized with *C. auris* or 2) used or touched equipment or shared space with an individual who is colonized or infected with *C. auris*.
General recommendations	In this document, *general recommendations* refer to existing guidance from public health authorities and professional societies to prevent *C. auris* in healthcare settings, most of which are derived from adult populations, but are relevant for pediatric populations.
High-touch surfaces	Surfaces in healthcare and non-healthcare settings that are frequently touched or handled by patients, caregivers, visitors, children, or healthcare personnel (e.g., tables, counters, bed rails, bedside tables, sink handles, toilet seats, call buttons, doorknobs, light switches). Thus, high-touch surfaces have a higher likelihood of contamination and pathogen transmission[4,5].
Local IPC	HCP within healthcare facilities who develop and help implement evidence-based policies and practices to reduce the risk of healthcare-associated infections in patients, staff, and visitors.
Infected	Individual with signs and symptoms consistent with infection and a positive culture or molecular test for *C. auris*[6].
Neonatal intensive care unit (NICU)	Specialized unit for premature, low birthweight, or critically ill newborn infants needing intensive care.
Neonate	A newborn who is less than 28 days old.
Non-acute healthcare settings	Licensed healthcare facilities or services that provide ongoing, rehabilitative, long-term residential, or outpatient care rather than short-term, high-intensity inpatient care for acute illness or injury. Examples may include long-term care facilities, inpatient rehabilitation facilities, outpatient clinics, dialysis centers, and home healthcare services.
Non-healthcare congregate settings	Shared residential or community environments that are not considered licensed healthcare facilities in which multiple unrelated children, usually with their family members, reside, receive services, or spend extended time together in close proximity. Examples include Ronald McDonald Houses[7], schools, daycare centers, and group homes.
Non-medical equipment	Non-clinical items that are used in healthcare settings for recreational, educational, or supportive purposes (e.g., entertainment consoles, gaming stations, computers, toys, and age-appropriate activity materials). These frequently touched items require routine cleaning and disinfection with appropriate germicidal products per institutional protocols[8].
Plain language	Presentation of health information in clear, straightforward wording that is understandable to patients and families without the need for specialized medical knowledge. Plain language avoids jargon and technical terminology (or clearly explains this terminology when necessary), uses active voice and short sentences, and organizes information so that important messages and recommended actions are easy to identify and follow.
Public health partners	Local or state governmental public health agencies that conduct disease surveillance, receive reports of notifiable infections, and provide guidance and support to healthcare facilities for outbreak investigation and infection prevention and control.
Rooming-in	The practice of allowing primary caregivers and their children, including neonates and infants to remain together while the child is hospitalized[9].
Shared decision-making	A structured, collaborative process in which clinicians, pediatric patients (to the extent consistent with their developmental capacity and preferences), and parents or legal guardians work together to make healthcare decisions. Shared decision-making integrates clinical evidence with the patient's evolving autonomy, the parents' or guardians' authority and values, and the clinical judgment of the care team[10].
Shared spaces	Areas within healthcare settings that are not dedicated to a single individual or care episode and may be used by multiple individuals, including patients, caregivers, visitors, or

	healthcare personnel, (e.g., lounges, playrooms, breastfeeding rooms, diagnostic or therapeutic areas). Shared spaces present an increased risk for indirect contact and environmental transmission to patients.
Skin-to-skin care	The practice of placing the skin of neonates and infants in direct contact with the skin of their caregivers (chest-to-chest). The infant is typically naked or dressed only in a diaper to maximize the surface-to-surface contact and the dyad is covered with prewarmed blankets, leaving the infant's head exposed[9].
Standard Precautions	IPC measures applied to care of all patients in healthcare settings regardless of known presence of potential pathogens.<br><br>Elements of Standard Precautions include appropriate hand hygiene; use of gowns and gloves if contact with bodily fluids, respiratory secretions, or excretions is anticipated; use of face protection if splashes or sprays is anticipated; respiratory hygiene; safe disposal of sharps and safe injection practices; and cleaning and disinfection of equipment and the environment.
Terminal cleaning	Terminal cleaning refers to the process of cleaning and disinfection of the patient's room when patients with *C. auris* colonization or infection are discharged or of shared spaces, e.g., physical therapy areas, used by patients with *C. auris* colonization or infection. Terminal cleaning should start with shared equipment and common surfaces, then proceed to surfaces and items touched during patient care and finally proceed to surfaces and items directly touched by the patient[11].
Surveillance	Proactive testing of patients for colonization or infection with *C. auris*, usually patients who are high-risk and/or with known exposures to 1) another patient colonized or infected with *C. auris* or (2) potentially contaminated equipment or space shared with another patient with known colonization or infection with *C. auris*.
U.S. Environmental Protection Agency (EPA) List P	EPA-registered antimicrobial products that have demonstrated efficacy against *C. auris* and are recommended for environmental cleaning and disinfection in healthcare and other settings when *C. auris* is present[11,12].
Visitor	Visitors are individuals who enter a healthcare facility to provide social, emotional, or personal support to a patient and who are not part of the healthcare workforce and not receiving medical care themselves (e.g., family members, friends, or support persons)[1].

1. The Society for Healthcare Epidemiology of America (SHEA). Handbook for SHEA-Sponsored Guidelines and Expert Guidance Documents, Expert Guidance Documents, Consensus Statements, and Practice Statements. https://shea-online.org/wp-content/uploads/2024/12/2024-Handbook-Final-Updated-112624.pdf
2. CORHA. About C. auris: Resources, Guides, Reports, & Toolkits. Feb. 10, 2026. Updated Jul. 25, 2024. Accessed Feb. 10, 2026. https://corha.org/diseases-pathogens/c-auris
3. Spaulding EH. Chemical disinfection of medical and surgical materials. In: Lawrence C, Block SS, eds. *Disinfection, sterilization, and preservation*. Lea & Febiger; 1968:517-531.
4. Kenters N, Kiernan M, Chowdhary A, et al. Control of Candida auris in healthcare institutions: Outcome of an International Society for Antimicrobial Chemotherapy expert meeting. *Int J Antimicrob Agents*. Oct 2019;54(4):400-406. doi:10.1016/j.ijantimicag.2019.08.013
5. Ahmad S, Asadzadeh M. Strategies to Prevent Transmission of Candida auris in Healthcare Settings. *Curr Fungal Infect Rep*. 2023;17(1):36-48. doi:10.1007/s12281-023-00451-7
6. Centers for Disease Control and Prevention. Infection Control Guidance: *Candida auris*. Feb. 10, 2026. Updated Apr. 24, 2024. Accessed Feb. 10, 2026. https://www.cdc.gov/candida-auris/hcp/infection-control/index.html
7. Guzman-Cottrill JA, Blatt DB, Bryant KA, et al. SHEA practice update: infection prevention and control (IPC) in residential facilities for pediatric patients and their families. *Infect Control Hosp Epidemiol*. Nov 14 2024;46(1):1-24. doi:10.1017/ice.2024.124

8. Ernst KD, Committee On Hospital C. Resources Recommended for the Care of Pediatric Patients in Hospitals. *Pediatrics*. Apr 2020;145(4)doi:10.1542/peds.2020-0204
9. Feldman-Winter L, Goldsmith JP, Committee On F, Newborn, Task Force On Sudden Infant Death S. Safe Sleep and Skin-to-Skin Care in the Neonatal Period for Healthy Term Newborns. *Pediatrics*. Sep 2016;138(3)doi:10.1542/peds.2016-1889
10. Eaton SM, Clark JD, Cummings CL, et al. Pediatric Shared Decision-Making for Simple and Complex Decisions: Findings From a Delphi Panel. *Pediatrics*. Nov 1 2022;150(5)doi:10.1542/peds.2022-057978
11. Centers for Disease Control and Prevention. Environmental Cleaning Procedures. Best Practices for Environmental Cleaning in Healthcare Facilities. Feb. 10, 2026. Updated Mar. 19, 2024. https://www.cdc.gov/healthcare-associated-infections/hcp/cleaning-global/procedures.html?CDC_AAref_Val=https://www.cdc.gov/hai/prevent/resource-limited/cleaning-procedures.html
12. US Environmental Protection Agency. EPA's Registered Antimicrobial Products Effective Against *Candida auris*. https://www.epa.gov/pesticide-registration/epas-registered-antimicrobial-products-effective-against-candida-auris-list
