# Supplementary material for: Infection prevention and control of Candida auris in pediatric settings
Source: Antimicrob Steward Healthc Epidemiol. 2026 Jun 23;6(1):e183. doi: 10.1017/ash.2026.10419 (PMC13312240; doi:10.1017/ash.2026.10419)
Supplement: Murray et al. supplementary material 3 — Murray et al. supplementary material [file S2732494X26104197sup003.pdf]

Supplementary Materials

Table 3. Knowledge Gaps

**Category**	**Insufficient evidence at time of publication**
Precautions	Duration of Contact Precautions for *C. auris* for infants and children for current and future admissions.
Precautions	Duration of Contact Precautions for infants and children with a caregiver who is colonized or infected with *C. auris*.
Precautions	Use of Contact Precautions for *C. auris* for future admissions if the child's screening cultures are negative in the setting of a caregiver or household member who is colonized with *C. auris*.
Screening	Relative yield of different body sites for *C. auris* screening.
Screening	Timing and frequency of screening children for *C. auris* who have ongoing, prolonged exposure to a caregiver who is colonized or infected with *C. auris*.
Screening	Timing and frequency of screening high-risk caregivers and household contacts expected to have repeated, long-term exposure to their child who colonized with *C. auris*, including their child's bodily fluids and skin.
Visitation	Visitation by siblings of pediatric patients with *C. auris* colonization or infection.
Decolonization	Agent and method effective for decolonization of *C. auris*.
Skin cleansing	Benefits or harms of breast cleansing before breastfeeding or skin cleansing before skin-to-skin contact by caregivers who are colonized or infected with *C. auris*.
Transmission risk	Risk of transmission of *C. auris* from the breast milk of caregivers colonized or infected with *C. auris*.
Transmission risk	Risk of *C. auris* transmission from neonates or infants with *C. auris* colonization or infection to parents or caregivers during breastfeeding or skin-to-skin practices.
Transmission risk	Risk of *C. auris* transmission or colonization in pediatric long-term care and rehabilitation facilities.
Transmission risk	Risk of direct patient-to-patient transmission of *C. auris* in pediatric non-acute healthcare settings.
Transmission risk	Risk of transmission of *C. auris* in non-healthcare congregate settings for children.
